# Supplementary material for: Genome-Wide Identification, Classification, and Expression Analysis of the HD-Zip Transcription Factor Family in Apple (Malus domestica Borkh.)
Source: Int J Mol Sci. 2022 Feb 27;23(5):2632. doi: 10.3390/ijms23052632 (PMC8910561; doi:10.3390/ijms23052632)
Supplement: Supplementary file 1 [file ijms-23-02632-s001.zip › ijms-1588271-supplementary.pdf]

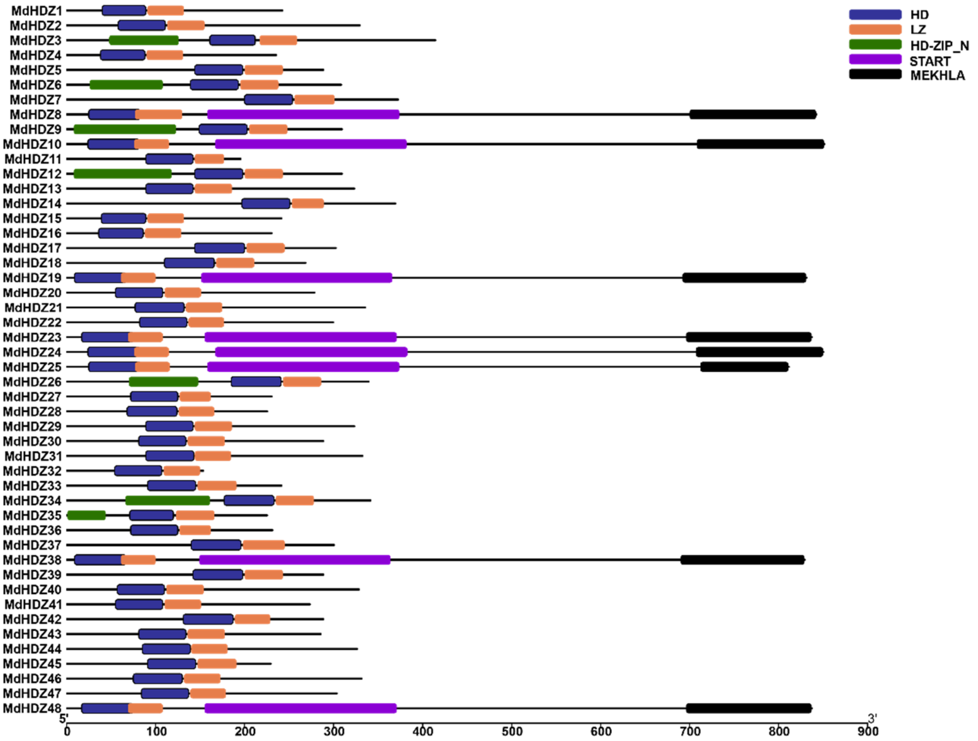

Figure S1. Conserved domains analysis of MdHDZs

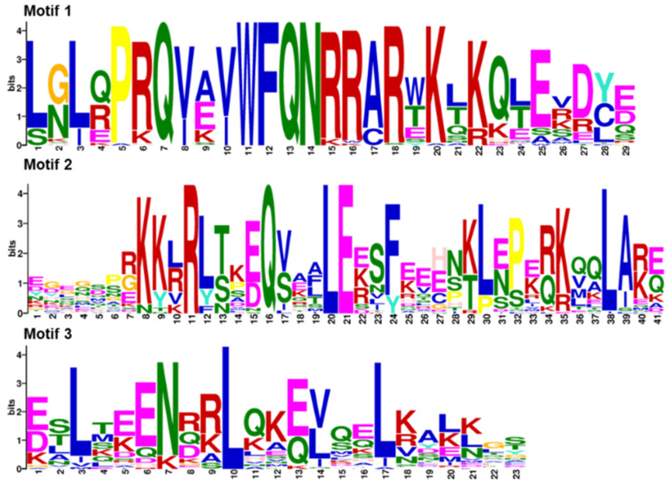

Figure S2. Sequence logos of 3 conserved motifs detected in the MdHDZs. The overall height of each stack represents the degree of conservation at this position, while the height of the individual letters within each stack indicates the relative frequency of the corresponding amino acid.

Table S1. Primer sequences used for qRT -PCR.

Gene Name	Forward primer (5′-3′)	Reverse primer (5′-3′)
MdHDZ1	CACACACGCACTCAGTTGTC	GATGGTGCTCTTGCCTGTGT
MdHDZ2	GTGAAGGTCGAGGAGCACAA	TGGATGGGACTGATGGAGTG
MdHDZ4	AGCATAAGTGAGGTGTGGACG	GGCTTACGCTTATCAAGCTGC
MdHDZ8	ACCATAAAGGAGGGCTTGGT	CACCCTCAATGAGATGGGCA

MdHDZ9	TTTGTTGGTGGGGAAAGGGAA	GCACGATCCTCCAATCCAATG
MdHDZ15	TGTAGGAGTTGGCATGTTGCT	TGTGCGTGTGTGTAAAAGGTC
MdHDZ16	CCCCGCACATAAAAGTTTGGT	GCTCAGAAGAATAAGACGCTGC
MdHDZ19	CCTCTTCTTTGCACCAAAGGC	AAACAAAGGGCAGCAGCATT
MdHDZ21	TCTCCGATGTCTTTGCAGCC	ACGTCGTTGAGGAAAACACT
MdHDZ22	CCGCATGAGATGAATGGAAAAA	AGGCACTGAAGATCTGACCG
MdHDZ23	AGCCGAAAGTGACGAACCAA	TGAAGGCTTTGGAGCTGGAG
MdHDZ24	TCATGGAGACTTTTCAGAGTGTT	TGACACCGCGTGAAACCTAA
MdHDZ31	CTGCCGCTCCATTTCTTTGC	TCCAATTAGGGACATGACGATTCA
MdHDZ34	AGTAGCAGAGAGAGCACCCA	CGGTTCAGACACAGCCAGAT
MdHDZ35	CGTGGTCACAACTGCCTCAG	GCTGCACCTTGACACTGCT
MdHDZ38	CCTCCAGCCATCCTTCTCAG	TTGTGTGAGCCAGGGGAAGA
MdHDZ40	CCCTGCTGGGTTGTGAAATC	CCCAAAATTGTGCCCAAAGGA
MdHDZ41	GGAGCAAAAATATCCAAGGTAAAGC	GCAGTTGCACCAAAAGTCCAA
MdHDZ42	TGTTGTCCTCTCTCCCCACTA	CCCTAATTGCAGGTGTGGGA
MdHDZ43	AGAGGCAACAAACCAAATTACCA	GCTGCAACACTCAGTCAGTC
MdHDZ48	CACTGATTCCACTCGCTCAG	CGGAGTTAGCACCAAACACA

Table S2. The numbers and genome size of *HD-Zip* homologous genes found in indicated species

Specie	Number of HD-ZIP	Genome Size (Mb)
Arabidopsis thaliana	47	125
Oryza sativa	33	430
Zea mays	55	2300
Triticum aestivum	46	17000
Setaria italica	47	490
Sesamum indicum	45	357
Solanum tuberosum	43	850
Glycine max	88	978
Malus domestica	49	687
Prunus persica	33	265
Cucumis sativus	40	367
Capsicum annuum	40	3070

Table S3. The duplication pairs of *MdHDZ* genes in apple

Segmental duplication pairs	
MdHDZ1	*MdHDZ15*
MdHDZ4	*MdHDZ16*
MdHDZ4	*MdHDZ15*
MdHDZ2	*MdHDZ20*
MdHDZ2	*MdHDZ41*
MdHDZ2	*MdHDZ40*
MdHDZ6	*MdHDZ12*
MdHDZ7	*MdHDZ14*
MdHDZ5	*MdHDZ17*
MdHDZ5	*MdHDZ37*

MdHDZ8	*MdHDZ25*
MdHDZ9	*MdHDZ12*
MdHDZ10	*MdHDZ24*
MdHDZ13	*MdHDZ31*
MdHDZ13	*MdHDZ44*
MdHDZ15	*MdHDZ16*
MdHDZ14	*MdHDZ34*
MdHDZ19	*MdHDZ23*
MdHDZ20	*MdHDZ41*
MdHDZ17	*MdHDZ37*
MdHDZ19	*MdHDZ38*
MdHDZ20	*MdHDZ40*
MdHDZ17	*MdHDZ39*
MdHDZ19	*MdHDZ48*
MdHDZ22	*MdHDZ30*
MdHDZ23	*MdHDZ38*
MdHDZ22	*MdHDZ43*
MdHDZ21	*MdHDZ46*
MdHDZ22	*MdHDZ47*
MdHDZ23	*MdHDZ48*
MdHDZ27	*MdHDZ36*
MdHDZ26	*MdHDZ34*
MdHDZ28	*MdHDZ42*
MdHDZ30	*MdHDZ43*
MdHDZ31	*MdHDZ44*
MdHDZ33	*MdHDZ45*
MdHDZ30	*MdHDZ47*
MdHDZ37	*MdHDZ39*
MdHDZ40	*MdHDZ41*
MdHDZ38	*MdHDZ48*
MdHDZ43	*MdHDZ47*

Tandem duplication pairs	
MdHDZ17	*MdHDZ18*
MdHDZ28	*MdHDZ29*
MdHDZ30	*MdHDZ31*
MdHDZ34	*MdHDZ35*
MdHDZ43	*MdHDZ44*

Table S4. 32 different organs/tissues apple transcriptome

Sample	Title
GSM1052621	Flower_M67 Replicate 1
GSM1052622	Flower_M67 Replicate 2
GSM1052623	Fruit_M74_100daa Replicate 1

GSM1052624	Fruit_M74_100daa Replicate 2
GSM1052625	Flower_M74 Replicate 1
GSM1052626	Flower_M74 Replicate 2
GSM1052627	Fruit_M20_100daa Replicate 1
GSM1052628	Fruit_M20_100daa Replicate 2
GSM1052629	Leaf_M14 Replicate 1
GSM1052630	Leaf_M14 Replicate 2
GSM1052631	Fruit_M20_harvest Replicate 1
GSM1052632	Fruit_M20_harvest Replicate 2
GSM1052633	leaf_M49 Replicate 1
GSM1052634	leaf_M49 Replicate 2
GSM1052635	Fruit_M74_harvest Replicate 1
GSM1052636	Fruit_M74_harvest Replicate 2
GSM1052637	Root_GD Replicate 1
GSM1052638	Root_GD Replicate 2
GSM1052639	Stem_X8877 Replicate 1
GSM1052640	Stem_X8877 Replicate 2
GSM1052641	Root_X8877 Replicate 1
GSM1052642	Root_X8877 Replicate 2
GSM1052643	Stem_GD Replicate 1
GSM1052644	Stem_GD Replicate 2
GSM1052645	Seedling_GD Replicate 1
GSM1052646	Seedling_GD Replicate 2
GSM1052647	Seed_X4442xX2596 Replicate 1
GSM1052648	Seed_X4442xX2596 Replicate 2
GSM1052649	Seedling_X4102 Replicate 1
GSM1052650	Seedling_X4102 Replicate 2
GSM1052651	Seed_X3069xX922 Replicate 1
GSM1052652	Seed_X3069xX922 Replicate 2

Table S5. Filtered reads quality statistics

Sample	Total Raw Reads (M)	Total Clean Reads (M)	Total Clean Bases(Gb)	Clean Reads Q20(%)	Clean Reads Q30(%)	Clean Reads Ratio(%)
HF_0H1	67.79	62.84	6.28	98.94	96.29	92.69
HF_0H2	67.79	62.65	6.27	99.03	96.69	92.42
HF_0H3	67.79	62.25	6.22	99.03	96.7	91.83
HF_18H1	67.79	60.95	6.09	98.97	96.48	89.91
HF_18H2	67.79	61.45	6.15	98.99	96.55	90.66
HF_18H3	67.79	61.36	6.14	99.06	96.77	90.52
HF_24H1	67.79	61.58	6.16	99.03	96.63	90.84
HF_24H2	67.87	62.83	6.28	98.97	96.57	92.58
HF_24H3	67.87	62.35	6.24	98.97	96.56	91.87
HF_48H1	67.87	62.49	6.25	98.94	96.48	92.07
HF_48H2	67.79	61.88	6.19	98.95	96.38	91.28
HF_48H3	67.79	62.29	6.23	99.02	96.56	91.89
HF_6H1	67.79	62.28	6.23	99.03	96.67	91.88
HF_6H2	69.97	62.69	6.27	98.92	96.34	89.59
HF_6H3	69.97	62.64	6.26	98.94	96.44	89.51

Table S6. Summary of RNA-seq and mapping results.

(1) Summary of mapping result (mapping to reference genome)			
Sample	Total Clean Reads (M)	Total Mapping(%)	Uniquely Mapping(%)
HF_0H1	62.84	81.82	63.1
HF_0H2	62.65	82.63	64.4
HF_0H3	62.25	81.63	62.66
HF_18H1	60.95	83.15	64.87
HF_18H2	61.45	83.82	68.48
HF_18H3	61.36	86.86	71.69
HF_24H1	61.58	83.87	65.42
HF_24H2	62.83	81.67	63.18
HF_24H3	62.35	82.85	63.6
HF_48H1	62.49	82.4	64.63
HF_48H2	61.88	83.85	65.27
HF_48H3	62.29	85.13	66.83
HF_6H1	62.28	82.92	64.17
HF_6H2	62.69	83.31	65.02
HF_6H3	62.64	84.09	66.42

(2) Summary of mapping result (mapping to reference genes)			
Sample	Total Clean Reads (M)	Total Mapping(%)	Uniquely Mapping(%)
GD_0H1	62.36	80.52	39.86
GD_0H2	62.28	82.85	41.59
GD_0H3	62.16	81.87	40.27
GD_18H1	61.07	81.41	41.7
GD_18H2	62.51	82.43	41.64
GD_18H3	61.24	83.15	43
GD_24H1	62.08	80.78	41.65
GD_24H2	60.85	81.33	41.8
GD_24H3	61.73	81.94	42.16
GD_48H1	60.88	80.06	42.07
GD_48H2	62.53	81.56	43.53
GD_48H3	62.53	80.21	42.7
GD_6H1	62.23	82.29	41.71
GD_6H2	62.14	81.5	41.91
GD_6H3	61.15	82.34	42.75
HF_0H1	62.84	78.55	39.68
HF_0H2	62.65	79.2	40.4
HF_0H3	62.25	78.49	39.97
HF_18H1	60.95	79.63	40.07
HF_18H2	61.45	79.6	39.31
HF_18H3	61.36	81.14	41.49
HF_24H1	61.58	79.7	39.86
HF_24H2	62.83	78.54	40.46
HF_24H3	62.35	79.69	41.15
HF_48H1	62.49	78.49	41.38
HF_48H2	61.88	79.95	40.91
HF_48H3	62.29	81.09	41.36

HF_6H1	62.28	79.62	41.03
HF_6H2	62.69	79.36	40.09
HF_6H3	62.64	79.99	41.07
